# Editorial: The Functional and Neural Mechanisms of Numerosity Processing: From Perception to Cognition

**DOI:** 10.3389/fnhum.2022.880645

**Published:** 2022-04-07

**Authors:** Michele Fornaciai, Joonkoo Park, Roberto Arrighi

**Affiliations:** ^1^Department of Cognitive Neuroscience, International School for Advanced Studies (SISSA), Trieste, Italy; ^2^Department of Psychological and Brain Sciences, University of Massachusetts Amherst, Amherst, MA, United States; ^3^Commonwealth Honors College, University of Massachusetts Amherst, Amherst, MA, United States; ^4^Department of Neuroscience, Psychology, Pharmacology and Child Health, University of Florence, Florence, Italy

**Keywords:** numerosity perception, numerical cognition, subitizing, groupitizing, mathematical abilities, perceptual adaptation, spatial-numerical association, connectedness

Perceiving numerosity—the number of discrete items in a set—represents a fundamental step to understand the surrounding environment. Its ubiquitous nature across animal species suggests that it provides important advantages for survival. It is also thought to serve as an important basis for advanced mathematical thinking in humans. The diverse nature of the perceptual and cognitive functions linked to numerosity has attracted a large number of researchers with different perspectives, methodologies, and levels of investigation. In line with this, we present here a series of ten articles capturing the multifaceted nature of numerosity perception and numerical cognition.

First, some of the contributions aimed to achieve a deeper understanding of the brain mechanisms tuned to numerosity by leveraging on perceptual illusions and/or contextual effects.

For example, Li et al. investigated numerosity perception in the periphery of the visual field, an area of particular interest due to its lower spatial “resolution” and the tendency to pool information across larger spatial extents. The results indicate that numerosity estimates for a given target area are robustly distorted by irrelevant contextual information in the surrounding areas, with the relative weight of these two sources of information depending on the position participants deployed their attention to.

Numerosity is related to space not only when it comes to central vs. peripheral vision, but also in terms of how quantities are mapped to spatial location (i.e., along a “mental number line”) and to spatial extent (i.e., the coupling of numerical and spatial magnitude). Viarouge and de Hevia addressed the interaction between these two types of mapping, showing that they may arise from a single representational system.

Another powerful tool to study numerosity perception is adaptation. Here, Togoli and Arrighi leveraged on this technique to show that adaptation generalizes not only across vision and audition but also touch, despite this latter modality exploits a completely different reference frame (hand/body centered). Haptic numerosity adaptation is indeed able to strongly distort perceived numerosity presented visually and auditorily, bolstering the idea that numerosity processing is modality independent.

Adaptation is not the only process that can bias numerosity perception. Numerosity is indeed intertwined with several continuous magnitudes (e.g., area, density) that could potentially interfere with it. Castaldi et al. addressed the influence of non-numerical attributes in numerosity perception in the context of working memory (WM) resources. When WM resources are deprived, numerosity perception becomes more vulnerable to interference, suggesting that WM plays a role in preventing non-numerical attributes from biasing numerosity perception.

Another interesting perceptual distortion is the “connectedness” illusion, in that connecting pairs of items in a display strongly reduces perceived numerosity. Is this connectedness illusion an automatic, passive process, or does it involve an active segmentation? Pomè et al. show that connectedness requires attentional resources, suggesting that this form of perceptual organization is likely an active process.

Grouping of visual elements (for example by connecting pairs of items) is not always detrimental to numerosity perception. Indeed, clustering a visual array into small groups (i.e., no more than 4 items) improves the discrimination of numerosity–an effect called “groupitizing.” Is groupitizing a purely visual mechanism, or does it involve an amodal mechanism as in the case of adaptation? Anobile et al. addressed this question, showing that the clustering of an auditory sequence of tones in small sub-groups significantly improves numerosity discrimination.

This groupitizing phenomenon however relies on another important mechanism, which is the *exact* estimation of very small (≤ 4) numerosities. This mechanism is called “subitizing” and is in contrast with the approximate estimation of larger numerosities. Fu et al. used EEG to investigate the encoding of approximate and subitizable numerosities during memory retention, showing a clear difference in their signatures. Interestingly, the signature of small numerosity processing resembles the typical pattern of EEG activity observed in WM tasks, indicating the role of WM in subitizing.

The study of the brain mechanisms involved in numerosity perception is also important in light of robust evidence that it is closely linked to higher-level cognitive functions. For example, formal mathematical abilities have been often observed to correlate with approximate numerosity estimation, suggesting a potential role of numerosity perception as a precursor of mathematics. Here, Tokita and Hirota addressed the relation between approximate numerosity and numeracy in adults across different numerosity judgement tasks. The results show that approximate numerical abilities are significantly related to numeracy irrespective of task, consistent with the idea that there exist overlapping processing mechanisms between numerosity and math.

Moreover, Ma et al. further investigated the resilience of the link between numerosity and math to auditory sensory deprivation (i.e., early deafness). Similarly to vision, the results show that this link holds even in the absence of the auditory input, suggesting that the relation between math and numerosity develops in a way that is independent of any specific sensory modality.

Finally, Szkudlarek et al. investigated the intuitive mathematical abilities of children prior to the actual acquisition of mathematical knowledge. To do so, the authors tested the ability to perform approximate divisions using numerosity stimuli, finding that even children that could not perform simple divisions were still able to do this perceptually-driven divisive operation. These findings suggest that this form of “intuitive arithmetic” precedes mathematical education, and it could represent a mechanism mediating the relationship between numerosity and math.

Overall, this Article Collection not only provides an overview of the multifaceted fields of numerosity perception and numerical cognition but also provides novel insights into the mechanisms of numerosity processing and its relationship with mathematical abilities. The many findings reported in this collection point to three overarching ideas: (1) although rooted in low-level perception, numerosity processing recruits amodal mechanisms abstracted from sensory processing; (2) numerosity processing likely involves an active mechanism requiring attentional and WM resources as well as top-down inputs; and (3) numerosity perception and intuitive arithmetic abilities are likely related to mathematical abilities during development and in adulthood.

## Author Contributions

All authors listed have made a substantial, direct, and intellectual contribution to the work and approved it for publication.

## Funding

This work was supported by the European Union's Horizon 2020 Research and Innovation Programme under the Marie Sklodowska-Curie grant agreement No. 838823 NeSt to MF and by the Italian Ministry of Education University and Research under the PRIN2017 program (Grant No. 2017XBJN4F-EnvironMag) to RA.

## Conflict of Interest

The authors declare that the research was conducted in the absence of any commercial or financial relationships that could be construed as a potential conflict of interest.

## Publisher's Note

All claims expressed in this article are solely those of the authors and do not necessarily represent those of their affiliated organizations, or those of the publisher, the editors and the reviewers. Any product that may be evaluated in this article, or claim that may be made by its manufacturer, is not guaranteed or endorsed by the publisher.

